# Pediatric Desmoplastic Fibroma of the Jaws: A Comprehensive Review of Clinical Presentation, Management, and Outcomes

**DOI:** 10.3390/diagnostics16111580

**Published:** 2026-05-22

**Authors:** George Batshon, Murad Abdelraziq, Imad Abu El-Naaj, Yasmine Ghantous

**Affiliations:** Tzafon Medical Center, Department of Oral and Maxillofacial Surgery, Affiliated with Azrieli Faculty of Medicine, Bar Ilan University, Ramat Gan 15208, Israel

**Keywords:** bone tumors, desmoplastic fibroma, fibromatosis, jaw tumors, mandible, maxillofacial, pediatric, recurrence, review, surgical resection

## Abstract

**Background:** Desmoplastic fibroma (DF) is a rare, benign, but locally aggressive intraosseous tumor with a predilection for the mandible in pediatric patients. Owing to its low incidence, evidence guiding management remains limited. **Objective:** To provide a comprehensive review of the clinical presentation, radiographic features, treatment strategies, and outcomes of pediatric DF of the jaws. **Methods:** A comprehensive literature review was conducted using PubMed/MEDLINE, Embase, Cochrane Library, and IEEE Xplore to identify relevant studies published between 2000 and 2026. Given the rarity of this entity, a broad search strategy was applied. Eligible studies were analyzed to extract data on patient demographics, clinical features, imaging findings, treatment modalities, and outcomes. **Results:** A total of 32 studies comprising 45 pediatric cases were identified. The mandible was involved in 86.7% of cases. The most common presentation was painless swelling or facial asymmetry (68.9%). Wide or segmental resection was the primary treatment in 68.9% of cases. Recurrence data were available for 75.6% of cases, with an overall recurrence rate of 2.9%, occurring following incomplete resection. **Conclusions:** Pediatric DF of the jaws is a rare but locally aggressive tumor requiring accurate diagnosis and individualized surgical management. Complete resection with clear margins appears to provide the most reliable outcomes. However, interpretation of outcomes is limited by the predominance of case reports, heterogeneous reporting, and incomplete follow-up. Future multicenter studies and standardized reporting are needed to better define optimal management strategies.

## 1. Introduction

Desmoplastic fibroma (DF) is an exceedingly rare, benign, yet locally aggressive intraosseous neoplasm that was first described by Jaffe in 1958 as the osseous counterpart of desmoid-type fibromatosis [1,2,3,4,5,6,7]. Although histologically benign, DF is characterized by infiltrative growth, progressive bone destruction, and a marked propensity for local recurrence following conservative surgical management [8,9,10,11,12]. The tumor accounts for less than 0.1% of all primary bone neoplasms and approximately 0.3% of benign osseous lesions, underscoring its exceptional rarity [1,2,3,4,5,6,7,8,9,10,11,12]. Despite its low incidence, DF poses a significant clinical challenge due to its aggressive local behavior and diagnostic overlap with other benign and malignant jaw lesions [1,2,3,4,5].

Within the craniofacial skeleton, DF demonstrates a pronounced predilection for the jaws, particularly the mandible. Up to 22% of all reported desmoplastic fibromas arise in the maxillofacial region, with the posterior mandibular body, angle, and ramus being most commonly involved [12,13,14,15,16,17,18,19,20,21,22]. Pediatric patients represent a substantial proportion of reported cases, with many lesions diagnosed during the first and second decades of life, including early childhood [1,2,3,4,5,6,7,8,12,13,14,15,16,17,18,19,20,21,22,23,24,25]. This age distribution highlights the importance of DF as a diagnostic and therapeutic consideration in children presenting with expansile mandibular lesions [12,13,14,15].

Clinically, pediatric DF typically presents as a slowly progressive, painless swelling leading to facial asymmetry. Due to its indolent course, lesions are often advanced at the time of diagnosis. Radiographic and histopathologic features are frequently nonspecific, with potential overlap with other benign and malignant jaw lesions, making accurate diagnosis challenging [26,27,28,29,30,31,32,33,34,35,36,37,38,39,40,41,42,43,44,45,46].

Surgical excision remains the mainstay of treatment for desmoplastic fibroma. Conservative approaches such as simple curettage or enucleation have been associated with recurrence rates ranging from 40% to 70%, whereas wide resection with histologically clear margins significantly reduces the likelihood of local recurrence [20,21,22,23,24,25,26,27,28,29,30,31,32,33,34,35,36].

The extreme rarity of pediatric desmoplastic fibroma of the jaws has resulted in a literature dominated by isolated case reports and small case series, limiting the availability of high-level evidence and standardized treatment guidelines. Nevertheless, the accumulating body of published data consistently emphasizes the importance of early diagnosis, meticulous surgical planning, and multidisciplinary management involving pediatric maxillofacial surgeons, pathologists, radiologists, and reconstructive specialists. A comprehensive synthesis of the existing literature is therefore essential to clarify the clinicopathologic spectrum of this entity, evaluate treatment outcomes, and inform evidence-based management strategies in affected children.

Pediatric patients present unique clinical challenges that distinguish them from adults, particularly regarding the impact of treatment on craniofacial growth, dental development, facial symmetry, and long-term functional outcomes. In addition, reconstructive strategies must account for ongoing skeletal growth and future rehabilitation needs. These considerations are not fully addressed in the broader literature on desmoplastic fibroma, which predominantly includes mixed-age or adult populations, supporting the need for a focused pediatric analysis.

Accordingly, the aim of this review is to analyze and integrate all available published data on pediatric desmoplastic fibroma of the jaws, with particular focus on clinical presentation, radiologic features, histopathologic and molecular characteristics, treatment approaches, and recurrence patterns, in order to support informed clinical decision-making and optimize long-term outcomes in this rare but challenging condition.

## 2. Methods

A comprehensive literature review was conducted to identify studies reporting desmoplastic fibroma of the jaws in pediatric patients. Electronic databases including PubMed/MEDLINE, Embase, Cochrane Library, and IEEE Xplore were searched using a combination of Medical Subject Headings (MeSH) and free-text terms. In addition, a supplementary search of Google Scholar was performed to identify potentially relevant grey literature and additional case reports not indexed in the primary databases. IEEE Xplore was included to ensure comprehensive coverage of interdisciplinary literature, particularly studies related to imaging techniques and diagnostic technologies relevant to craniofacial pathology. The search strategy focused on the primary disease term:

(“desmoplastic fibroma”[Title/Abstract] OR “desmoplastic fibroma”[MeSH Terms]).

No additional restrictions regarding anatomical location or patient population were applied at the search stage, as these factors are often inconsistently reported in titles and abstracts. Instead, eligibility criteria relating to craniofacial involvement (jaw/maxilla/mandible) and pediatric age group were applied during the screening process. Retrieved articles were reviewed for relevance based on titles and abstracts, followed by full-text evaluation where appropriate. The reference lists of included articles were also manually reviewed to identify any additional relevant studies not captured in the initial search. No language restrictions were applied during the initial search phase. However, only English-language publications were included in the final analysis due to resource constraints.

### 2.1. Inclusion and Exclusion Criteria

#### 2.1.1. Inclusion Criteria

Studies were eligible for inclusion if they met the following criteria:

- Original research articles, case reports, or case series describing desmoplastic fibroma involving the mandible or maxilla;
- Studies including patients younger than 18 years, or mixed-age studies with extractable pediatric data;
- Articles reporting clinical presentation, radiologic findings, histopathologic features, treatment approach, and/or follow-up outcomes;
- Studies providing sufficient detail to confirm the diagnosis of desmoplastic fibroma;
- Full-text articles available in English.

#### 2.1.2. Exclusion Criteria

Studies were excluded if they met any of the following criteria:

- Review articles, editorials, conference abstracts, letters, or expert opinions without original patient data;
- Studies describing desmoplastic fibroma exclusively in extra-gnathic skeletal sites without jaw involvement;
- Adult-only studies with no extractable pediatric data;
- Reports lacking adequate clinical, radiologic, or histopathologic confirmation of desmoplastic fibroma;
- Duplicate publications or overlapping patient cohorts (in such cases, the most comprehensive or recent report was included).

### 2.2. Study Selection Process

Retrieved articles were reviewed for relevance based on titles and abstracts, followed by full-text evaluation where appropriate. Data were extracted from eligible studies, including demographic characteristics, clinical presentation, imaging findings, treatment modalities, and outcomes. The following variables were extracted from each included study, when available: age, sex, anatomical location, presenting symptoms, imaging findings, histopathology, treatment modality, follow-up duration, and recurrence. Because desmoplastic fibroma of the jaws is an extremely rare entity, the available literature consists primarily of case reports and small case series, which are predominantly indexed in PubMed/MEDLINE.

Due to the nature of the available literature, which consists predominantly of case reports and small case series, formal quality assessment tools and systematic review elements—such as PRISMA guidelines and risk-of-bias assessment—were not applied, as they are not well-suited for evaluating isolated case-based evidence. Instead, the available data were summarized descriptively, with emphasis on clear and transparent reporting of findings.

## 3. Results

A total of 45 pediatric cases of desmoplastic fibroma (DF) of the jaws published between 2000 and 2026 were identified and included in this comprehensive review. These cases were extracted from individual case reports and small case series originating from multiple geographic regions including North America, Europe, Asia, the Middle East, and South America. The clinical, radiologic, therapeutic, and histopathologic characteristics of the included cases are summarized in Table 1.

### 3.1. Patient Demographic Characteristics

**Figure 1:**
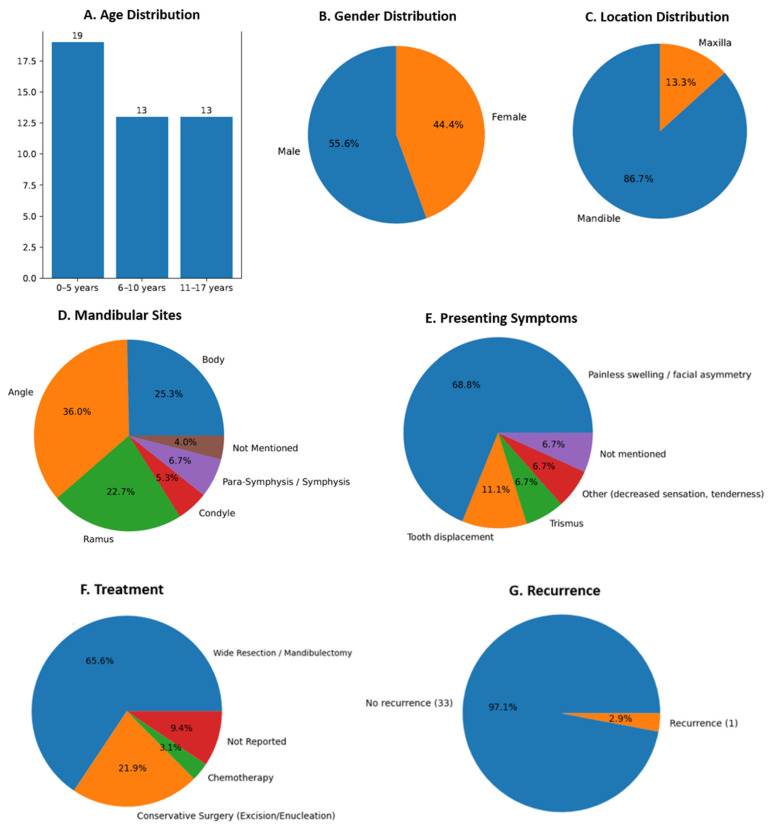
Summary of demographic, anatomical, and clinical characteristics of pediatric desmoplastic fibroma of the jaws. (**A**) Age distribution of patients (n = 45). (**B**) Gender distribution of included cases. (**C**) Anatomical distribution of lesions between mandible and maxilla. (**D**) Distribution of mandibular tumor sites (n = 39). Subsite involvement was not mutually exclusive; frequencies are reported rather than percentages; some lesions involved more than one mandibular region. (**E**) Clinical presentation, showing the relative frequency of reported symptoms (n = 45). (**F**) Treatment modalities utilized across included cases. (**G**) Recurrence outcomes following different treatment approaches.

The age of patients ranged from 6 months to 17 years, with a mean age of approximately 7.6 years and a median age of approximately 6 years. The majority of cases occurred within the first decade of life, supporting the recognized predilection of desmoplastic fibroma for pediatric and adolescent populations. Among the 45 patients, 25 were male (55.6%) and 20 were female (44.4%), indicating a slight male predominance. Figure 1A illustrates the age distribution of the included patients, demonstrating that desmoplastic fibroma of the jaws predominantly affects children across a broad pediatric age range. Figure 1B shows the gender distribution of the cases, indicating a relatively balanced occurrence between male and female patients. These findings emphasize that DF may present even during infancy and early childhood, in both genders.

### 3.2. Anatomical Distribution

The mandible was the most frequently involved site, accounting for 39 cases (86.7%), whereas the maxilla was involved in 6 cases (13.3%). Within the mandible, lesions most commonly involved the posterior regions, including the body, angle, and ramus. Figure 1C depicts the anatomical distribution of lesions, with a clear predominance of mandibular involvement compared to the maxilla. The distribution of mandibular tumor sites is shown in Figure 1D. The angle and body of the mandible were the most frequently involved regions, followed by the ramus, para-symphyseal/symphyseal region, and condyle. These findings are reported as frequencies of involvement rather than percentages of patients, as some lesions involved multiple mandibular subsites.

### 3.3. Clinical Presentation

The most common presenting complaint was painless swelling or facial asymmetry, which occurred in 68.9% of reported cases (31 cases). Other clinical manifestations included tooth displacement or dental involvement in 11.1% of reported cases, trismus in 6.7% of reported cases. Pain was uncommon, supporting the generally slow-growing and painless nature of DF despite its locally aggressive behavior. Figure 1E summarizes the clinical presentation, with painless swelling or facial asymmetry being the most common presenting symptom, followed by less frequent features such as tooth displacement and trismus.

### 3.4. Radiographic and Imaging Findings

Radiographic evaluation was performed using panoramic radiography (OPG), computed tomography (CT), magnetic resonance imaging (MRI), and occasionally ultrasound (US). The most common radiographic appearance was an expansile radiolucent lesion, identified in 33 cases (73.3%). Other common radiographic characteristics included multilocular radiolucency reported in 12 cases (26.7%), soap-bubble or trabeculated appearance in 7 cases (15.6%), and cortical expansion or destruction in 21 cases (46.7%). MRI findings commonly demonstrated low signal intensity on T1-weighted images and variable hyperintensity on T2-weighted sequences.

### 3.5. Treatment Modalities

Management strategies were primarily surgical, although the extent of resection varied. Wide or segmental resection was the most commonly reported treatment and was performed in 31 cases (68.9%). Conservative surgical approaches, including enucleation or local excision, were reported in 10 cases (22.2%). Reconstruction of mandibular defects was required in 16 cases (35.6%), most frequently using costochondral rib grafts, free fibula flaps, or reconstruction plates. Chemotherapy was used in 3 cases (6.7%), generally as an adjunctive treatment modality. Figure 1F outlines the treatment modalities, demonstrating that surgical resection—particularly wide or segmental resection—was the most commonly employed approach.

### 3.6. Recurrence and Follow-Up

Among cases with available follow-up data, follow-up periods ranged from 6 months to 17 years. Recurrence data were available for 34 of 45 cases (75.6%). In 11 cases (24.4%), recurrence status was not reported. Among cases with reported follow-up, tumor recurrence was documented in one patient (2.9%), which occurred after incomplete initial surgical removal. The patient subsequently underwent definitive resection with reconstruction, with no further recurrence reported. These findings reinforce the importance of complete surgical resection with clear margins to reduce recurrence risk. Figure 1G illustrates recurrence outcomes, showing that recurrence was uncommon overall and was primarily associated with cases managed with conservative or incomplete surgical treatment.

### 3.7. Histopathological Analysis

Histopathologic examination across the included cases demonstrated consistent features characteristic of desmoplastic fibroma. These included spindle-shaped fibroblasts arranged in fascicles within a dense collagenous stroma, low cellularity, and minimal mitotic activity. Immunohistochemical analysis, when performed, frequently demonstrated positive staining for vimentin and β-catenin.

## 4. Discussion

This review provides a comprehensive overview of the clinical presentation, imaging characteristics, management strategies, and outcomes of pediatric desmoplastic fibroma (DF) of the jaws. To the best of our knowledge, this review represents one of the largest syntheses of pediatric DF of the jaws reported in the literature between 2000 and 2026, comprising 45 pediatric cases identified from published case reports and small case series.

The findings highlight the predominance of mandibular involvement, the typical clinical presentation of painless swelling or facial asymmetry, and the importance of complete surgical resection to minimize recurrence.

### 4.1. Predilection for the Mandible

One of the most consistent findings in the literature is the strong predilection for the mandible, which accounted for 39 of 45 cases (86.7%) in the present review.

This observation is consistent with previous studies reporting that the mandible represents the most common craniofacial site affected by DF [17,23].

Within the mandible, the posterior regions—particularly the body, angle, and ramus—were the most frequently involved sites. Analysis of the available descriptions of lesion location indicated that the mandibular angle (36%) and body (25.3%) were the most commonly affected regions, followed by the ramus (22.7%), while involvement of the para-symphyseal and condyle region was rare for both sites (6.7%). These findings are reported as frequencies of involvement rather than percentages, as several lesions involved multiple mandibular subsites.

Several hypotheses have been proposed to explain this anatomical distribution. The posterior mandible contains a larger volume of cancellous bone, which may provide a favorable microenvironment for fibroblastic proliferation. Additionally, the mandible develops through intramembranous ossification derived from neural crest cells, which may influence the biological behavior of fibroblastic tumors in the craniofacial skeleton [16]. The posterior mandibular body and angle regions also experience greater biomechanical stress, which may contribute to tumor initiation or progression [16,17].

In contrast, maxillary involvement was uncommon, accounting for 6 of 45 cases (13.3%). When present, maxillary lesions often produced earlier clinical symptoms because of the proximity to surrounding anatomical structures such as the nasal cavity, maxillary sinus, and orbit, as demonstrated in cases reported by Cupero [1] and Fahmy et al. [21].

### 4.2. Clinical Presentation and Growth Behavior

The most common presenting symptom identified in this review was painless swelling or facial asymmetry, which occurred in the majority of reported cases (68.9%). This presentation reflects the slow growing but infiltrative nature of DF, allowing the lesion to expand within bone before producing significant symptoms [17]. Other clinical manifestations observed in the reviewed cases included tooth displacement or dental involvement, cortical expansion, facial asymmetry, and trismus. These findings are consistent with previous studies describing DF as a locally aggressive tumor capable of extensive bone remodeling and cortical expansion [20].

Pain was rarely reported, likely due to the tumor’s tendency to expand within cancellous bone without early involvement of neural structures. However, when lesions become sufficiently large to involve surrounding musculature or the temporomandibular joint region, functional limitations such as trismus may develop, as described by Kadowaki et al. [22].

### 4.3. Radiographic Characteristics and Diagnostic Challenges

Radiographically, DF most commonly appears as an expansile radiolucent lesion, which may be either unilocular or multilocular. In several cases, lesions demonstrated trabeculated or “soap-bubble” patterns, closely resembling other odontogenic tumors such as ameloblastoma or odontogenic myxoma [9,10].

Advanced imaging modalities play an essential role in evaluating tumor extent. Computed tomography (CT) is particularly useful for identifying cortical destruction and soft tissue extension, while magnetic resonance imaging (MRI) provides superior soft tissue contrast and can demonstrate the internal fibrous composition of the lesion [5,15].

Despite the use of advanced imaging techniques, the radiographic appearance of DF remains nonspecific, which may lead to misdiagnosis or delayed diagnosis. The differential diagnosis often includes Ameloblastoma, Odontogenic myxoma, Fibrous dysplasia, Central giant cell granuloma, and Low-grade fibrosarcoma [8,9,10,11,12,13,14,15]. Therefore, histopathological examination remains essential for definitive diagnosis.

This diagnostic overlap often results in delayed or inaccurate initial diagnosis, emphasizing the importance of a multidisciplinary approach integrating clinical, radiologic, and histopathological findings. Early biopsy and careful correlation between imaging and histologic features are essential to establish an accurate diagnosis and guide appropriate management.

### 4.4. Histopathological and Molecular Features

Histologically, DF is characterized by spindle-shaped fibroblasts arranged in interlacing fascicles within a dense collagenous stroma. The lesions typically demonstrate low cellularity, minimal nuclear atypia, and rare mitotic figures, distinguishing them from malignant spindle-cell neoplasms [6,7,8,9,10,11,12,13,14,15,16,17].

Immunohistochemical studies frequently show vimentin positivity, consistent with the tumor’s mesenchymal origin. Several studies have also demonstrated β-catenin expression, suggesting the involvement of the Wnt signaling pathway, similar to desmoid-type fibromatosis [20,21,22,23,24,25,26,27,28,29,30,31,32,33,34,35].

Recent molecular studies further support this association. For example, Kadowaki et al. [22] reported a CTNNB1 mutation in a case of mandibular DF, strengthening the molecular link between DF and desmoid-type fibromatosis. However, some authors have proposed that DF of the jaws may represent a distinct entity, given the neural crest origin of craniofacial bones, which differs from the mesodermal origin of most skeletal sites [16].

### 4.5. Treatment Strategies and Surgical Considerations

Surgery remains the cornerstone and mainstay of treatment for desmoplastic fibroma of the jaws due to its infiltrative growth pattern and capacity for progressive osseous destruction. Across the 45 pediatric cases included in the present review, wide resection or segmental mandibulectomy was the most frequently reported treatment approach, accounting for 31 of 45 cases (68.9%), whereas more conservative procedures such as enucleation or local excision were used less frequently.

Historically, conservative approaches—including curettage and enucleation—were attempted in an effort to preserve surrounding structures. However, these techniques have been associated with significantly higher recurrence rates, reported to range from 40% to 70% [17,33]. In contrast, current evidence supports wide surgical resection with adequate margins as the most reliable method for reducing recurrence risk. This approach was consistently favored in several cases included in the present review, including those reported by Skinner et al. (2017) [19], Karimi et al. (2020) [24], and Andrade et al. (2024) [29], as well as in other studies demonstrating extensive mandibular involvement or soft-tissue extension.

The choice of surgical procedure appears to depend largely on lesion extent, cortical perforation, soft-tissue involvement, and the anticipated morbidity of resection. Smaller, well-contained lesions were occasionally managed with conservative surgery, whereas larger tumors involving the mandibular body, angle, or ramus were more commonly treated with marginal, segmental, or hemimandibulectomy. This pattern is consistent with previous reports [6,15,19,20,24,29,32], all of which support wider resection in cases demonstrating aggressive radiologic or clinical features.

Although conservative treatment may preserve anatomical structures and reduce immediate reconstructive morbidity, incomplete excision remains the most significant risk factor for recurrence. In the present review, the only clearly documented recurrence occurred in the case reported by Mohammadi et al. [25] following prior incomplete treatment, whereas subsequent segmental mandibulectomy achieved disease control. This finding reinforces the importance of achieving adequate initial surgical margins, particularly in lesions with ill-defined radiologic or histopathologic boundaries.

However, surgical management in pediatric patients presents unique challenges. Radical resection may adversely affect mandibular growth centers, dental development, occlusion, and facial symmetry. Therefore, treatment planning should not be reduced to a simple dichotomy between conservative and radical approaches. Instead, surgical decisions should be individualized, considering tumor behavior, anatomical extent, patient age, and reconstructive considerations, ideally within a multidisciplinary framework.

### 4.6. Reconstruction in Pediatric Patients

Reconstruction of mandibular defects after tumor ablation remains one of the most demanding aspects of management in children. In the present review, reconstruction was reported in 16 of 45 cases (35.6%), reflecting the frequency with which extensive mandibular lesions required immediate structural restoration. Reported reconstructive approaches included costochondral or rib grafts, reconstruction plates, and vascularized fibula free flaps. Rib-based reconstruction was used in several earlier reports, including those by Hereford et al. [2], Ferri et al. [15], and Khatib and Pogrel [20], whereas plate-based reconstruction was reported by Mohammadi et al. [25], Karimi et al. [24], and Gonçalves et al. [28]. Vascularized fibula reconstruction was described in more recent and more extensive defects by Skinner et al. [19], Andrade et al. [29], and Batshon et al. [32].

This evolution in reconstructive choice is clinically meaningful. Non-vascularized rib or costochondral grafts remain attractive in selected children because they are technically simpler and may provide a biologically familiar option for ramus-condyle reconstruction. However, their limitations include resorption, non-union, unpredictable growth behavior, and the potential need for secondary procedures, as illustrated in some long-term pediatric mandibular reconstructions. In contrast, the fibula free flap offers a long, straight, well-vascularized segment of bone capable of restoring continuity in major mandibular defects, while also providing a stable platform for future functional rehabilitation. Batshon et al. [32] emphasize that in extensive pediatric mandibular DF, fibula reconstruction can restore contour and continuity reliably, particularly when combined with meticulous planning and long-term follow-up.

The available pediatric DF literature does not yet establish a single superior reconstructive strategy for all patients, but it does suggest that vascularized fibula reconstruction is especially valuable for long-segment defects involving the body and ramus. Skinner et al. [19] reported durable function and symmetry at six years after fibula free flap reconstruction, while Andrade et al. [29] described successful immediate reconstruction using a double-barrel fibula flap in a 5-year-old child. Batshon et al. [32] further showed that fibula-based reconstruction, combined with virtual surgical planning and patient-specific guides, can achieve accurate restoration of mandibular contour and stable early outcomes even in aggressive, extensive disease.

An important point, however, is that vascularized fibula does not reproduce native mandibular growth potential. As Batshon et al. [32] discuss, and as broader pediatric reconstructive principles suggest, the fibula flap should be viewed primarily as a stable structural scaffold, not as a growth-equivalent substitute for the developing mandible. For this reason, children reconstructed with fibula flaps require prolonged surveillance throughout growth, with the expectation that secondary contouring, augmentation, occlusal adjustment, or implant-related rehabilitation may be needed later.

Virtual surgical planning also deserves emphasis. In extensive mandibular defects, 3-dimensional planning, cutting guides, and pre-bent fixation plates improve the precision of osteotomies and facilitate more accurate restoration of contour, mandibular height, and condylar position. Batshon et al. [32] incorporated these principles in their case, and their discussion supports the view that advanced digital planning may reduce technical error and improve reconstructive predictability in complex pediatric cases.

Taken together, the reviewed literature suggests a practical reconstructive framework: rib or costochondral grafts may remain appropriate for selected smaller or ramus-condylar defects, whereas vascularized fibula free flap appears more suitable for extensive body–angle–ramus resections requiring immediate, durable restoration of continuity. Plate-only reconstruction may serve as a temporizing or selected definitive option, but in growing children it does not provide biologic replacement and may require later revision.

Importantly, the choice between reconstructive strategies should not be based solely on defect size, but also on anticipated long-term functional and esthetic outcomes in the growing child. While vascularized fibula reconstruction provides reliable structural stability and allows for future dental rehabilitation, it lacks intrinsic growth potential and may require secondary procedures during skeletal maturation. In contrast, costochondral grafts offer theoretical growth potential and may be advantageous in selected ramus-condylar reconstructions, although their growth behavior is unpredictable and may result in asymmetry [32].

Therefore, the decision between conservative versus radical resection and the choice of reconstructive technique must be individualized, balancing oncologic control with preservation of function, facial symmetry, and long-term craniofacial development. Given the limited quality of available evidence, no single reconstructive approach can be considered universally superior, underscoring the need for long-term follow-up and multidisciplinary treatment planning [32].

### 4.7. Recurrence and Prognosis

Overall, recurrence in pediatric jaw DF appears to be uncommon when adequate resection is achieved, although the published evidence remains limited by incomplete follow-up reporting. In the present review, 33 of 45 cases (73.3%) had no reported recurrence, 1 case (2.2%) had documented recurrence, and 11 cases (24.4%) did not report recurrence status. When only cases with follow-up data are considered, the recurrence rate remains low, but this should be interpreted cautiously given the retrospective and case-based nature of the literature.

The clearest pattern across studies is that recurrence is associated primarily with incomplete or conservative initial treatment, rather than with any single anatomical site or age group. This is consistent with the observations of Woods et al. [17], Khatib and Pogrel [20], and Batshon et al. [32], all of whom emphasize the importance of clear margins and prolonged postoperative surveillance.

From a prognostic standpoint, most children achieved good local control and acceptable functional or aesthetic outcomes, especially when treatment combined appropriate oncologic resection with thoughtful reconstruction and long-term follow-up. Accordingly, pediatric DF of the jaws should be approached not only as a benign fibro-osseous neoplasm, but also as a condition requiring longitudinal craniofacial management extending into adolescence and, in some cases, adulthood.

While incomplete or conservative treatment appears to be associated with increased recurrence risk, this observation should be interpreted with caution given the limited follow-up data and the case-based nature of the available evidence.

### 4.8. Diagnostic Considerations and Differential Diagnosis

Accurate diagnosis of desmoplastic fibroma (DF) of the jaws is essential but challenging, owing to its rarity and its overlap with other benign and malignant fibro-osseous and mesenchymal lesions [1,2,3,4,5,6,7,8,9,10,11,12]. The diagnostic process relies on a combination of clinical presentation, radiographic evaluation, and definitive histopathological assessment.

Clinically, DF most commonly presents as a slow-growing, painless swelling of the jaw, frequently resulting in facial asymmetry. In pediatric patients, this insidious presentation may delay diagnosis until significant cortical expansion or facial deformity becomes evident. Pain, paresthesia, or functional impairment are uncommon but may occur in advanced lesions with cortical perforation or soft-tissue extension [20,21,22,23,24,25,26,27,28,29].

Radiographically, DF typically appears as a unilocular or multilocular radiolucent lesion with well-defined or occasionally ill-defined borders. [15,16,17,18,19,20,21,22,23,24,25,26,27,28,29,30]. Despite these imaging characteristics, radiographic findings are not pathognomonic, and DF may closely resemble several other jaw lesions. Therefore, histopathological examination remains the gold standard for diagnosis. [30,31,32,33,34,35].

The differential diagnosis of DF is broad and includes both benign and malignant entities. Among benign lesions, odontogenic tumors such as ameloblastoma and odontogenic myxoma may present with similar radiographic features, particularly when multilocular. Central giant cell granuloma may also appear as an expansile radiolucent lesion in younger patients. Fibrous dysplasia and ossifying fibroma should be considered in cases with mixed radiographic patterns, although these lesions typically exhibit different growth dynamics and histologic features [25,26,27,28,29,30,31,32,33,34,35,36,37,38,39,40].

The histopathological diagnosis of desmoplastic fibroma may be challenging and requires differentiation from other spindle cell lesions of the jaws [21,22,23,24,25,26]. Key differential diagnoses include low-grade fibrosarcoma, which demonstrates increased cellular atypia and mitotic activity; fibrous dysplasia, characterized by irregular woven bone within a fibrous stroma; and desmoid-type fibromatosis of soft tissue. Additional entities such as myofibroma and low-grade fibromyxoid sarcoma may also be considered. Distinction from low-grade fibrosarcoma is particularly important, as it represents a malignant entity with overlapping histologic features but typically exhibits greater cellular atypia, higher mitotic activity, and more aggressive clinical behavior. Correlation with clinical, radiologic, and immunohistochemical findings is essential for accurate diagnosis [21,22,23,24,25,26]. Immunohistochemical analysis can aid in the diagnostic process, particularly in challenging cases. DF often shows positivity for β-catenin, reflecting its relationship to fibromatosis, although this finding is not entirely specific. Correlation with clinical and radiographic findings is therefore essential to establish an accurate diagnosis [22,23,24,25,26,27,28,29,30].

Given the potential for misdiagnosis and the implications for treatment, a multidisciplinary approach involving radiologists, pathologists, and maxillofacial surgeons is critical. Early and precise diagnosis enables appropriate surgical planning, which is essential for achieving local control while minimizing functional and developmental morbidity in pediatric patients [30,31,32,33,34].

### 4.9. Strengths of Available Evidence

Despite the rarity of pediatric desmoplastic fibroma of the jaws, this study provides a comprehensive synthesis of the available literature. To the best of our knowledge, this review represents one of the most extensive compilations of pediatric cases reported between 2000 and 2026, allowing for a more detailed characterization of the clinical presentation, imaging features, management strategies, and outcomes associated with this rare tumor.

A major strength of this study is the inclusion of detailed clinical variables—including patient demographics, tumor location, radiographic characteristics, treatment modalities, reconstructive approaches, and recurrence outcomes—allowed for a comprehensive comparison across published reports.

Another important strength is the quantitative synthesis of clinical trends, including anatomical distribution, presenting symptoms, treatment strategies, and recurrence rates, which provides clinicians with a clearer understanding of the disease behavior and optimal management strategies.

Finally, by integrating previously published cases with the newly reported case by Batshon et al. [32], this review contributes additional clinical evidence supporting the role of radical surgical resection with appropriate reconstruction as the most reliable treatment strategy for achieving long-term disease control in pediatric patients.

### 4.10. Limitation of Available Evidence

Despite the insights provided by this review, several important limitations must be acknowledged. The available evidence is derived predominantly from isolated case reports and small case series, which inherently limit the ability to perform robust statistical analyses and reduce the generalizability of the findings. In addition, heterogeneity in the reporting of clinical presentation, imaging characteristics, treatment approaches, and follow-up duration complicates direct comparison across studies and weakens the strength of the conclusions.

Inconsistent reporting of long-term follow-up and recurrence outcomes further limits accurate assessment of disease behavior and may lead to underestimation of true recurrence rates. Moreover, the absence of standardized treatment protocols across institutions makes it difficult to directly compare conservative and radical surgical approaches or establish definitive management guidelines.

Collectively, these limitations—including data heterogeneity, incomplete follow-up, and the predominance of case-based evidence—warrant cautious interpretation of the findings and significantly restrict their generalizability. Variability in reporting and the lack of standardized outcome measures further hinder the development of evidence-based treatment recommendations.

These challenges underscore the need for future research focused on multicenter collaboration and the establishment of prospective registries to enable standardized data collection, improve long-term outcome reporting, and support the development of more robust, evidence-based management strategies for pediatric desmoplastic fibroma of the jaws. Nevertheless, the overall consistency of clinical, radiographic, and histopathological findings across the included studies supports the reliability of the observed patterns.

## 5. Conclusions

This review highlights the characteristic clinical and radiographic features of pediatric desmoplastic fibroma of the jaws, including its strong predilection for the mandible and its typical presentation as painless swelling or facial asymmetry. Radiographically, the lesion most often appears as an expansile radiolucency associated with cortical expansion. Although histologically benign, desmoplastic fibroma exhibits locally aggressive and infiltrative behavior within bone, with the potential for significant osseous destruction if left untreated.

The findings of this review support complete surgical resection with clear margins as the most reliable treatment strategy, as recurrence is strongly associated with incomplete excision and conservative surgical approaches. In cases requiring extensive mandibular resection, reconstructive options such as costochondral grafts or vascularized free fibula flaps have demonstrated satisfactory functional and aesthetic outcomes, even in growing pediatric patients.

Early recognition and accurate diagnosis are critical to optimizing management. Comprehensive evaluation, including appropriate imaging and histopathological confirmation, combined with multidisciplinary surgical planning, is essential to achieve disease control while preserving mandibular growth, function, and facial symmetry. Given the rarity of this tumor, long-term follow-up and collaborative multicenter studies are necessary to further refine treatment protocols and strengthen the evidence base.

Future research integrating molecular and genetic insights with long-term clinical and reconstructive outcomes may further enhance our understanding and management of pediatric desmoplastic fibroma of the jaws.

Clinically, the key take-home message is that desmoplastic fibroma of the jaws in pediatric patients should be considered in the differential diagnosis of expansile mandibular lesions, and early diagnosis combined with complete surgical resection remains essential to minimize recurrence while preserving long-term function, facial symmetry, and craniofacial growth.

### 5.1. Clinical Implications

Although rare, desmoplastic fibroma should be considered in the differential diagnosis of expansile radiolucent lesions of the jaws in pediatric patients, particularly when lesions involve the posterior mandible and demonstrate progressive cortical expansion. Early diagnosis is essential because DF, despite its benign histology, exhibits locally aggressive behavior with potential cortical destruction and soft tissue invasion [17,23].

Radiologic evaluation using CT and MRI plays a crucial role in assessing tumor extent and surgical planning, especially when lesions involve critical anatomical structures such as the mandibular canal, masticatory muscles, or skull base [15,18]. However, imaging alone is insufficient for definitive diagnosis due to overlap with other odontogenic and non-odontogenic lesions.

From a therapeutic standpoint, the findings of this review reinforce that complete surgical resection with adequate margins is the most reliable method for preventing recurrence, particularly in large or aggressive lesions. In pediatric patients, treatment planning must balance oncologic control with preservation of mandibular growth, occlusion, and facial symmetry.

### 5.2. Future Research Directions

Given the rarity of pediatric desmoplastic fibroma of the jaws, further research is needed to better understand its biological behavior, optimal treatment strategies, and long-term outcomes. Most currently available evidence is derived from single case reports or small case series, highlighting the need for multicenter collaboration and prospective case registries.

Future investigations should aim to clarify the molecular and genetic mechanisms underlying DF, particularly the role of β-catenin signaling and CTNNB1 mutations, which have been identified in several reported cases [22,25]. Improved understanding of these pathways may provide insight into tumor pathogenesis and potential targeted therapies.

Additionally, long-term studies evaluating growth outcomes and functional rehabilitation after mandibular reconstruction in pediatric patients are needed. As reconstructive techniques continue to evolve, particularly with the increasing use of microvascular free tissue transfer, further research should assess how these interventions influence craniofacial development, dental rehabilitation, and quality of life in affected children.

Overall, while the clinical and therapeutic patterns identified in this review are consistent with previously reported findings, the current evidence base remains limited. The rarity of the condition, combined with the predominance of case reports, underscores the need for cautious interpretation and highlights the importance of future studies aimed at improving the quality and consistency of available data. Ultimately, the development of standardized treatment guidelines and reporting protocols would facilitate more consistent data collection and improve evidence-based management of this rare but clinically significant tumor.

## Figures and Tables

**Table 1:** Summary of demographic, clinical presentation, imaging findings, management, and outcomes of the 45 pediatric cases of desmoplastic fibroma of the jaws included in this review (n = 45, 2000–2026).

Author (Year)	Origin	Site	Sex	Age	Clinical Presentation	Imaging	Treatment	Follow-Up & Recurrence
Cupero et al. 2001 [1]	USA	Max.	F	14	Painless swelling, tooth displacement	Homogenous lesion of the right maxilla (CT, MRI)	Maxillectomy	>2 years, no recurrence
Hereford et al. 2001 [2]	USA	Mand.	F	11	Painless swelling of the left mandibular angle region.	Circumscribed, radiolucent-radiopaque lesion (OPG)	Marginal mandibulectomy, with costochondral rib graft	Not reported
Levin et al. 2003 [3]	USA	Mand.	M	13	Painless swelling of left buccal cortex	OPG & CT—radiolucent lesion	Chemotherapy followed by mandibular resection with free fibular bone graft reconstruction	Not reported
Vargaz-Gonzales et al. 2004 [4]	Mexico	Mand.	M	14	Painless swelling	CT—aggressive bone tumor with trabeculated, soap bubble appearance	Not reported	Not reported
Wippold II et al. 2005 [5]	USA	Mand.	F	0.5	Painless swelling and facial asymmetry	Expansile lytic lesion in the right hemimandible (US, CT, MRI)	Mandibulectomy	Not reported
Said-Al-Naief et al. 2006 [6]	UK	Mand.	M	8	Painless swelling and facial asymmetry	Disruption of the right inferior cortex of the mandible (OPG, CT)	Resection with wide margins	4.5 years, no recurrence
Sandrini et al. 2007 [7]	Brazil	Mand.	M	11	Painless swelling and facial asymmetry	Expandable growth in the left mandibular angle area (OPG)	Surgical removal of the lesion	33 months, no recurrence
Iatrou et al. 2008 [8]	Greece	Mand.	M	10	Trismus	Poorly circumscribed radiolucent lesion in the left angle region of the mandible (OPG, CT, MRI)	Excision, immediate restoration with an autogenous iliac crest free bone graft	5 years, no recurrence
Summa et al. 2010 [9]	Italy	Mand.	F	3	Painless hard swelling	Poly-lobulated defined mass in the right mandible metaphysis, osteolytic (CT, MRI)	Chemotherapy and tumor resection	Not reported
Kalia et al. 2011 [10]	India	Mand.	M	17	Facial asymmetry, painless swelling of left angle and body of mand.	Irregular radiolucent radio-opaque lesion (OPG, CT)	Enucleation	6 months, no recurrence
Scott et al. 2011 [11]	UK	Mand.	F	10	Facial asymmetry with contralateral midline shift	Cystic enlargement at the left mand. angle (CT)	Enucleation	3.5 years, no recurrence
Shekhar et al. 2011 [12]	India	Mand.	M	10	Painless swelling of angle	Unilocular radiolucent lesion with well-defined Margins (OPG, CT)	Surgical excision and curettage	7 months, no recurrence
Tandon & Garg 2012 [13]	India	Max.	F	8	Painless buccal swelling	Well-defined osteolytic lesion (OPG, CT)	Excision	Not reported
Azola et al. 2012 [14]	USA	Max.	M	1.5	Painless swelling	Well-circumscribed, expansile, lucent lesion (CT)	Wide excision	Every 2 months, no recurrence
Ferri et al. 2013 [15] Case 1	Italy	Mand.	F	3	Painless swelling	Mass of high contrast enhancement and vascular flow-void (MRI, CT)	Partial right mandibulectomy, reconstruction using costochondral grafts	36 months, no recurrence
Ferri et al. 2013 [15] Case 2	Italy	Mand.	F	2	Painless swelling	MRI & CT—lesion involving the left mandible	Partial mandibulectomy, reconstruction with costochondral grafts	26 months, no recurrence
Ferri et al. 2013 [15] Case 3	Italy	Mand.	F	2	Progressive swelling of the right mandible.	Extensive mass (CT)	Mandibular resection, reconstruction with costochondral grafts	17 years, no recurrence
Flucke et al. 2014 [16] Case 1	Netherlands	Mand.	F	8	Osseous lesion, not further reported.	Not reported	Excision	Not reported
Flucke et al. 2014 [16] Case 2	Netherlands	Mand.	M	3	Osseous lesion, not further reported.	Not reported	Resection with negative margins	13 years, no recurrence
Woods et al. 2015 [17]	USA	Mand.	F	13	Painless swelling, tooth displacement	Multilocular radiolucency (OPG)	Marginal resection	3.5 years, no recurrence
Gersak et al. 2015 [18]	Romania	Mand.	M	3	Facial asymmetry, painless swelling	Osteolytic, trabeculated lesion in body of the right mandible, ill-defined borders (OPG, US, CT)	Wide local resection	Not reported
Skinner et al. 2017 [19]	Chile	Mand.	M	3	Painless swelling of body and ramus right mandible	Expansive intraosseous mixed density lesion (CT)	Mandibulectomy, reconstruction with a free fibula flap	6 years, no recurrence
Khatib & Pogrel 2017 [20] Case 1	USA	Mand.	F	8	Painless swelling, tooth displacement	Well-circumscribed, expansile, lytic lesion of the mandibular body (OPG, CT)	Resection with 1 cm margins, immediate reconstruction with costochondral grafts	14 years, no recurrence
Khatib & Pogrel 2017 [20] Case 2	USA	Mand.	F	9	Not reported	Lesion in left ascending ramus, medial cortical perforation (OPG, CT, MRI)	Segmental resection and immediate reconstruction with costochondral grafts	13 years, no recurrence
Khatib & Pogrel 2017 [20] Case 3	USA	Mand.	M	2	Facial asymmetry, painless swelling	Mass on posterior body and ramus with lateral bony spicule (OPG, CT)	Segmental resection, reconstructed with a costochondral rib graft	12 years, no recurrence
Khatib & Pogrel 2017 [20] Case 4	USA	Mand.	F	2	Trismus	Osteolytic lesion in right mandibular ramus (CT, MRI)	Chemotherapy	18 years, no tumour progression
Fahmy et al. 2019 [21]	USA	Max.	F	12	Painless swelling	Bony expansion with irregular, well-circumscribed hypodense area (CT)	Resection with 1 cm clear margins	1 year, no recurrence
Kadowaki et al. 2020 [22]	Japan	Mand.	M	5	Painless left mandibular swelling; trismus, facial asymmetry	Multilocular radiolucency (OPG)	Surgical resection: molecular analysis (CTNNB1 mutation)	1 year, no recurrence
Kahraman et al. 2020 [23] Case 1	Turkey	Mand.	F	16	Painless swelling	Radiolucent lesion	Complete surgical excision.	No recurrence (Follow up not reported)
Kahraman et al. 2020 [23] Case 2	Turkey	Max.	M	17	Painless swelling	Expansive well demarcated radiolucent lesion, 3 cm in diameter	Complete surgical excision.	No recurrence (Follow up not reported)
Kahraman et al. 2020 [23] Case 3	Turkey	Bilateral Mand.	F	8	Painless swelling	Multilocular, expansive radiolucent lesion	Surgical excision	No recurrence (Follow up not reported)
Kahraman et al. 2020 [23] Case 4	Turkey	Mand.	F	12	Painless swelling	Multilocular large radiolucent lesion with tooth luxation	Surgical excision	No recurrence (Follow up not reported)
Kahraman et al. 2020 [23] Case 5	Turkey	Mand.	M	9	Painless swelling	Radiolucent lesion	Surgical excision	No recurrence (Follow up not reported)
Kahraman et al. 2020 [23] Case 6	Turkey	Mand.	M	4	Painless swelling	Radiolucent destructive lesion	Surgical excision	No recurrence (Follow up not reported)
Karimi et al. 2020 [24] Case 1	Iran	Mand.	F	2	Facial asymmetry, painless swelling of right angle	Lytic lesion with expansion of buccal and lingual cortical plates (CT)	Segmental mandibulectomy with 1 cm safety margin, reconstruction with plate	8 months, no recurrence
Karimi et al. 2020 [24] Case 2	Iran	Max.	M	9	Painless swelling	Large mass (MRI)	Partial maxillectomy	11 months, no recurrence
Karimi et al. 2020 [24] Case 3	Iran	Mand.	M	1.5	Facial asymmetry due to right mandibular swelling.	Radiolucent, well-defined, expansile lesion in the right posterior mandible (CT)	Mandibulectomy	3 years, no recurrence
Mohammadi et al. 2020 [25]	Iran	Mand.	M	2	Painless swelling in right mandible	Extensive lesion in ramus and angle of mand. (CT)	Enucleation, followed by segmental mandibulectomy and plate reconstruction	Recurrence within < 1 year. Post 2nd surgery—1.5 years, no recurrence
Nisha et al. 2021 [26]	India	Mand.	M	15	Painless swelling of body of the right mandible	Expansile lytic lesion in body of the mandible (OPG, CT)	En-bloc resection with a wide margin	4 years, no recurrence
Motevasseli et al. 2022 [27]	Iran	Mand.	M	5	Painless swelling on the right side of the mandible body	Lytic lesion on the lingual side of the right mandibular body with cortex destruction (OPG, US, CBCT)	Surgical resection with wide margins, reconstruction using a surgical plate	14 months, no recurrence
Gonçalves et al. 2022 [28]	Brazil	Mand.	M	17	Painless swelling in the right mand.	Extensive hypodense lesion (OPG, CT)	Block resection with 1 cm free margins and plate reconstruction	3 years, no recurrence
Andrade et al. 2024 [29]	India	Mand.	F	5	Painless swelling in angle of the mandible, tooth displacement	Ill-defined, multilocular osteolytic lesion (OPG, CBCT)	Segmental resection with safe margins, reconstruction with a double-barrel fibula free flap	4 years, no recurrence
Siddiqui et al. 2024 [30]	Pakistan	Mand.	M	6	Painless right facial swelling, facial asymmetry	Well-defined borders lesion with cortical expansion (OPG, CT)	Enucleation, reconstruction by placement of allogenic bone graft	1 year, no recurrence
Al-Khateeb et al. 2025 [31]	Jordan	Mand.	M	1.9	Painless swelling in the right mand. area	Heterogonous soft tissue with destruction of angle and body of mandible (US, CT)	Neoadjuvant chemotherapy followed by marginal resection	2.5 years, no recurrence
Batshon et al. 2026 [32]	Israel	Mand.	M	10	Painless swelling of right mandibular border	Expansile, osteolytic lesion, ill-defined, with cortical thinning and expansion (OPG, CT, MRI)	Segmental mandibulectomy, reconstruction with a free fibula graft	15 months, no recurrence

Abbreviations: Max. = Maxilla; Mand. = Mandible; OPG = orthopantomogram; CBCT = Cone Beam Computed Tomography; CT = computed tomography; MRI = magnetic resonance imaging; US = Ultrasound.

## Data Availability

The original contributions presented in this study are included in the article. Further inquiries can be directed to the corresponding author.
